# Interleukin-17 Induces an Atypical M2-Like Macrophage Subpopulation That Regulates Intestinal Inflammation

**DOI:** 10.1371/journal.pone.0108494

**Published:** 2014-09-25

**Authors:** Kenichiro Nishikawa, Naohiro Seo, Mie Torii, Nei Ma, Daisuke Muraoka, Isao Tawara, Masahiro Masuya, Kyosuke Tanaka, Yoshiyuki Takei, Hiroshi Shiku, Naoyuki Katayama, Takuma Kato

**Affiliations:** 1 Department of Hematology and Oncology, Mie University Graduate School of Medicine, Tsu, Mie, Japan; 2 Department of Immuno-Gene Therapy, Mie University Graduate School of Medicine, Tsu, Mie, Japan; 3 Department of Cellular and Molecular Immunology, Mie University Graduate School of Medicine, Tsu, Mie, Japan; 4 Faculty of Health Science, Suzuka University of Medical Science, Suzuka, Mie, Japan; 5 Gastroenterology and Hepatology, Mie University Graduate School of Medicine, Tsu, Mie, Japan; Charité, Campus Benjamin Franklin, Germany

## Abstract

Interleukin 17 (IL-17) is a pleiotropic cytokine that acts on both immune and non-immune cells and is generally implicated in inflammatory and autoimmune diseases. Although IL-17 as well as their source, mainly but not limited to Th17 cells, is also abundant in the inflamed intestine, the role of IL-17 in inflammatory bowel disease remains controversial. In the present study, by using IL-17 knockout (KO) mice, we investigated the role of IL-17 in colitis, with special focus on the macrophage subpopulations. Here we show that IL-17KO mice had increased susceptibility to DSS-induced colitis which was associated with decrease in expression of mRNAs implicated in M2 and/or wound healing macrophages, such as IL-10, IL-1 receptor antagonist, arginase 1, cyclooxygenase 2, and indoleamine 2,3-dioxygenase. Lamina propria leukocytes from inflamed colon of IL-17KO mice contained fewer CD11b^+^Ly6C^+^MHC Class II^+^ macrophages, which were derived, at least partly, from blood monocytes, as compared to those of WT mice. FACS-purified CD11b^+^ cells from WT mice, which were more abundant in Ly6C^+^MHC Class II^+^ cells, expressed increased levels of genes associated M2/wound healing macrophages and also M1/proinflammatory macrophages. Depletion of this population by topical administration of clodronate-liposome in the colon of WT mice resulted in the exacerbation of colitis. These results demonstrate that IL-17 confers protection against the development of severe colitis through the induction of an atypical M2-like macrophage subpopulation. Our findings reveal a previously unappreciated mechanism by which IL-17 exerts a protective function in colitis.

## Introduction

Inflammatory bowel disease (IBD) including ulcerative colitis (UC) and Crohn’s disease (CD) is a chronic inflammatory disease with recurring relapses and remissions in the lower gastrointestinal tract [1], [2]. Genetic, environmental factors and their interrelationships which trigger an overactive adaptive immune response to intestinal bacterial flora have been considered to play key roles in the pathogenesis of IBD. More recent evidence suggests that defects in mucosal innate immune functions may also be involved in the etiology of IBD. However the exact pathomechanisms of the disease are still not fully elucidated.

IL-17, a signature cytokine of Th17 cells, is a pleiotropic cytokine with a primarily proinflammatory function that induces the production of other downstream inflammatory cytokines and chemokines [3]. IL-17 is found in abundance in the sera and affected tissues of various inflammatory and autoimmune diseases such as rheumatoid arthritis, multiple sclerosis, systemic lupus erythematosus psoriasis, and asthma and is well documented for its role in the pathogenesis of these diseases. IL-17 as well as their source, mainly but not limited to Th17 cells, is also found in the inflamed intestine in both animal models and humans [4], [5], but the role of IL-17 in IBD remains controversial [6], [7]. In models of both acute and chronic intestinal inflammation where mice were administered with trinitrobenzene sulfonic acid or infected with *Helicobacter hepaticus*, respectively, IL-17 showed a pathogenic role [8], [9]. Likewise, antibody mediated neutralization of IL-17 in mice bearing a conditional deletion of Stat3 in Foxp3^+^ T cells ameliorated the spontaneous colitis developed in these mice [10]. On the other hand, in a T-cell transfer colitis model, transfer of IL-17^−/−^ T cells induced more severe colitis in Rag^−/−^ mice [11]. In a dextran sulfate sodium (DSS)-induced colitis model, IL-17 has been shown to exert pro- [12] and anti-[13], [14] colitogenic activities, adding an additional layer of complexity.

These contradictory results regarding pathogenic versus protective roles of IL-17 mentioned above led us to re-examine whether IL-17 exerts protective function in a DSS-induced colitis model, focusing on the phenotypic and functional differences between macrophages in inflamed colon of WT and IL-17KO mice. Our results demonstrate that CD11b^+^Ly6C^+^MHC Class II^+^ macrophages were reduced in the inflamed colon of IL-17KO mice that accompanied the development of more severe colitis as compared to WT mice. Depletion of CD11b^+^Ly6C^+^MHC Class II^+^ macrophages in colon of WT mice resulted in more severe colitis. In vivo transfer experiments indicate that IL-17 promotes monocyte differentiation into M2-like macrophages. These results indicate that IL-17 protects from the development of DSS-induced colonic inflammation likely by promoting induction of unique macrophage subpopulation with anti-inflammatory and/or tissue repair functions.

## Materials and Methods

### Mice

C57BL/6 mice, C57BL/6 (CD45.1) congeneic mice and IL-17A deficient (IL-17KO) mice (C57BL/6 background) [15] were fed a standard diet, housed under specific pathogen free conditions and used at 5–8 weeks of age. All animal experiments were conducted under protocols approved by the Animal Care and Use Committee of Mie University Life Science Center.

### Induction of colitis

Colitis was induced by 1.5% DSS (MW: 36,000–50,000, MP Biomedicals) dissolved in drinking water for 7 days followed by water alone. The body weight change was monitored daily up to 15 days. Healthy control animals received water only. In some experiments, DSS colitis was induced in mice adoptively transferred with monocytes or mice treated with clodronate liposomes. Briefly, monocytes (7×10^6^ cells) isolated from bone marrow were transferred via tail vein into a group of mice a day before start of DSS treatment. Another group of mice were injected with 100 µl of clodronate liposomes or control liposomes (FormuMax Scientific Inc.) intrarectally using a feeding needle on days −1, 1, 3, and 5 of DSS administration.

### Assay for intestinal permeability

To evaluate epithelial barrier function, intestinal permeability was assessed by administration of FITC-dextran as described [16]. Briefly, DSS treated mice were given FITC-dextran (MW: 4,000, Sigma-Aldrich, 40 mg/100 g of body weigh) by oral gavage 4 h before killing. The amount of FITC-dextran in serum was measured on spectrophotometer (Molecular Devices).

### Isolation of colonic lamina propria leukocytes

Colonic lamina propria lymphocytes were collected from colons as described [17] with some modifications. Briefly, colons were resected and flushed with PBS to remove luminal contents. Colons were then opened longitudinally and cut into 0.5 cm pieces. The colonic pieces were incubated with HBSS without Ca^2+^ and Mg^2+^ containing 2.5% FCS, 1 mM DTT (Wako Pure Chemical Industries, Ltd. Japan), and 1% Penicillin-Streptomycin-Glutamine (Gibco) by shaking (200 rpm) at 37°C for 15 min to remove mucus. Subsequently, epithelial cells were removed through incubation with HBSS containing 2.5% FCS, 1mM EDTA, and 1% Penicillin-Streptomycin-Glutamine by shaking (200 rpm) at 37°C for 30 min. The colonic pieces were then digested in HBSS containing 2.5% FCS, 1.5 mg/ml Collagenase VIII (Sigma-Aldrich), and 0.1 mg/ml DNase I (Worthington Biochemical Corporation) by shaking (200 rpm) at 37°C for 30 min. Resultant cell suspensions were passed sequentially through cell strainers (100 µm), resuspended in 40% Percoll (GE Healthcare) and layered over an 75% Percoll prior to centrifugation at 2,500 rpm for 20 min. Cells from 40%/75% interface were collected, washed with HBSS for three times and used for experiments.

### Isolation of monocytes

Monocytes were isolated from bone marrow with EasySep mouse monocyte enrichment kit (StemCell Technologies, Vancouver, CA), according to the manufacturer’s instructions. Briefly, bone marrow cells from femora and tibiae were labeled with a cocktail of biotinylated antibodies against a panel of antigens expressed on T, B, NK, DCs, progenitor cells and granulocytes, followed by anti-biotin microbeads. Unlabeled monocytes were sorted magnetically by negative selection. Monocyte population contained more than 82% CD11b^+^Ly6C^+^ cells and less than 8% Ly6G^+^ cells, and used for in vivo transfer experiments.

### Flow cytometry

Colonic LP cells and monocytes were incubated with anti-CD16/32 (24G2; eBioscience) to block non-specific FcR followed by cell surface staining with corresponding mixture of fluorescently labeled mAbs. 7AAD (BD Pharmingen) was used to exclude nonviable cells. The following antibodies conjugated with FITC, PE, APC, V450, or APC/Cy7 were used for flow cytometry: anti-CD45.2 (104), anti-CD45.1 (A20), anti-I-Ab (AF6-120.1), anti-Ly6C (HK1.4), anti-F4/80 (BM8), anti-Ly6G (IA8), anti-CD206 (C068C2), anti-CD64 (X54-5/7.1), anti-CD127 (A7R34), anti-TCRγ/δ (GL3) (all from BioLegend), CD36 (NO. 72-1), anti-CCR9 (CW-1.2), anti-CCR7 (4B12), anti-CD11c (N418), anti-CD4 (RM4-5) (all from eBioscience), anti-CD11b (M1/70), Sca-1 (D7) (BD Bioscience), anti-CCR2 (475301) (R&D systems). PE-Rat IgG2a (eBR2a; eBioscience) and APC/Cy7-RatIgG2a (RTK 2758; BioLegend) were used as isotype matched control Abs. Data were acquired on a FACScant II (BD) and processed by using FlowJo software (Tree Star) with appropriate isotype controls to determine gating.

### Gene expression analysis

Total RNA was extracted from distal colon segments or purified cells with Trizol reagent (Invitrogen) and reverse transcribed into cDNA as described [18]. Realtime RT-PCR was performed by using FastStart Universal SYBR Green Master (Roche Diagnostics) according to the manufacturer’s instruction. Expression of target mRNA were normalized to the expression of β-actin mRNA for generation of ΔCt values, and relative mRNA expression was quantified with the ΔΔCt method [19]. Primer sequences for these reactions were designed by using Primer Express software Version 3 (Applied Biosystems) and provided in the Table S1.

### Histology

Distal colon tissue was fixed in 10% paraformaldehyde and embedded in paraffin blocks. Five micrometer sections were stained with hematoxylin and eosin.

### Statistics

Statistic analyses were performed with IBM SPSS Statistics Software Version 19 (IBM). Differences between two groups were compared using the two-tailed Student’s t-test and those among multiple groups were compared using the Kruskal-Wallis with Bonferroni post hoc’ test. A p value of <0.05 was considered significant. All experiments were performed more than two times. Data are presented as mean ± SEM.

## Results

### IL-17-deficient mice exhibit more severe acute colitis following DSS administration

**Figure 1:**
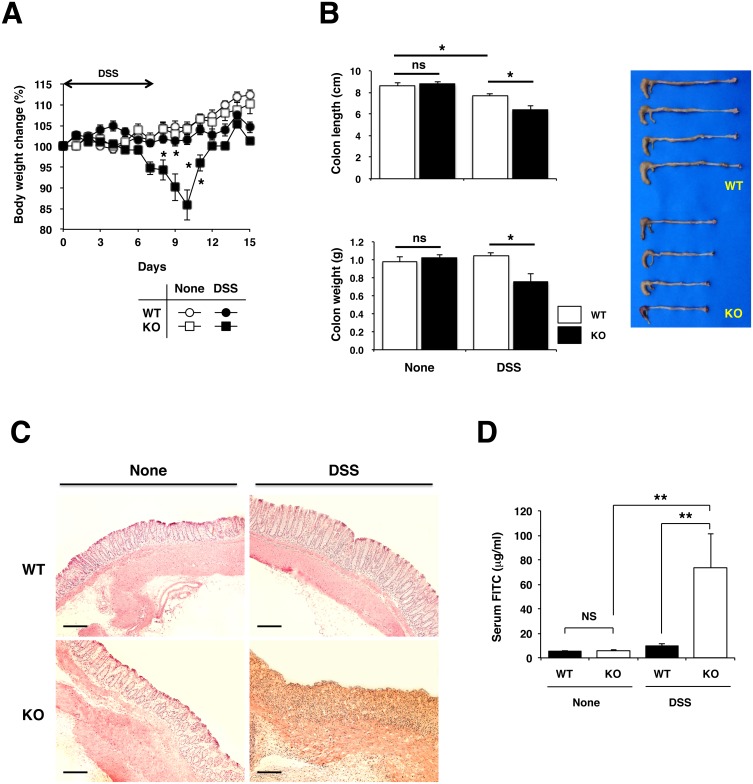
IL-17 deficient mice exhibit more severe acute colitis following DSS administration. IL-17KO mice and WT controls were given 1.5% DSS in drinking water or water alone for 7 days followed by consumption of water alone. Colitis severity was assessed by weight loss (A), colon length (day 10) (B) and H&E histology (day 0 and 10) (Calibration bar = 200 µm) (C). Colon barrier permeability was assessed on day 10 by detection of FITC-dextran serum (D). The results are expressed as mean values ± SEM for each geneotype in A (n = 5–6), B (n = 10), C (n = 2), and D (n = 5). *p<0.05; **p<0.01.

To reassess the role of IL-17 in DSS-induced colitis, we administered 1.5% DSS in drinking water to age- and sex-matched IL-17KO and WT mice for 7 days followed by water consumption alone with untreated mice serving as controls. At a steady state, both genotypes displayed no gross signs of colitis such as growth retardation, weight loss or diarrhea. Although ingestion of DSS is known to cause intestinal inflammation in WT mice as a result of disruption of the gut epithelial barrier, this dose of DSS did not induce marked weight loss in WT mice (Figure 1A). By sharp contrast, IL-17KO mice experienced significant weight loss starting at day 7, reaching a maximum reduction (15%) on day 10, followed by a recovery that reached a pretreatment level at day 5 after DSS withdrawal. In addition, IL-17KO mice showed a more decrease in colon length and weight by day 10 as compared to WT mice (Figure 1B). Histological analysis also revealed aggravated colonic inflammation as evidenced by edema, high degree of ulcerations and overt inflammatory infiltrate in both mucosa and submucosa, and mucosal thickening accompanied by destruction of epithelium in IL-17KO mice (Figure 1C). Concomitant with these findings, epithelial barrier function in IL-17KO mice was more severely compromised by DSS administration than that in WT mice as IL-17KO mice displaying a dramatic increase in orally administered FITC-dextran translocation into the serum (Figure 1D). Although WT mice treated with DSS did not exhibit overt symptoms of progressive wasting disease, we observed an increased number of inflammatory infiltrates and IL-17^+^ cells in the colon of WT mice treated with DSS as compared to the colons of untreated WT mice (Figure 1C and data not shown).

### Inflamed colons of IL-17KO mice expressed reduced levels of mRNA with anti-inflammatory functions

**Figure 2:**
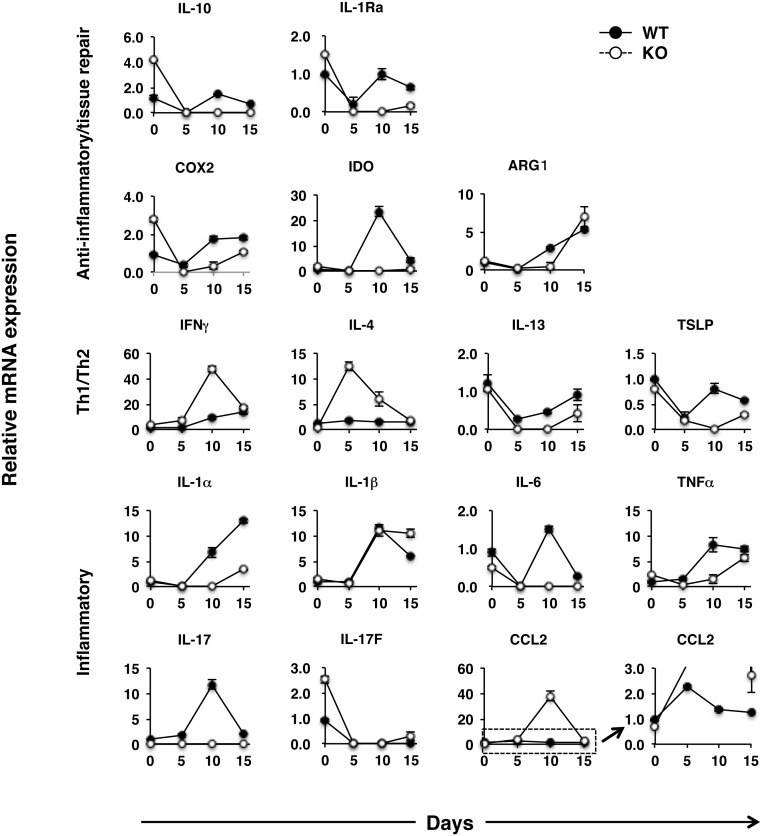
Colonic expression of mRNA for genes involved in inflammation and anti-inflammation. IL-17KO mice and WT controls were treated with DSS as described in the legend for Figure 1. Total RNA were purified from distal colon sections of untreated and DSS-treated individual mice (n = 3) and transcribed into cDNA, which were subsequently subjected for real-time PCR analysis. Expression of target mRNA were normalized to the expression of β-actin mRNA for generation of ΔCt values, and relative mRNA expression was quantified with the ΔΔCt method. Data are expressed as mean ± SEM. *p<0.05; **p<0.01.

Using real-time PCR samples taken from the colons of WT and IL-17KO mice, we next assessed the expression of mRNA for a range of genes thought to be involved in inflammatory/anti-inflammatory responses. mRNA for IL-10, IL-1 receptor antagonist (IL-1Ra), arginase 1 (ARG1), cyclooxygenase 2 (COX2), and indoleamine 2,3-dioxygenase (IDO), which are produced by M2 and/or wound healing macrophages contributing to the suppression/resolution of inflammation and tissue repair [20], were significantly reduced in IL-17KO mice at the peak of colon inflammation (day 10), as compared to WT mice (Figure 2). Notably, among mRNA for IL-22, DARC, and D6, all of which also have tissue repair and anti-inflammatory functions and are produced by a variety of cell types other than macrophages [21], [22], only DARC mRNA was significantly reduced in IL-17KO mice (data not shown). As for expression levels of Th1 and Th2 signature cytokines, the expression of IFN-γ and IL-4 mRNA were significantly upregulated in IL-17KO mice with severe colitis. Those for other inflammatory cytokines, such as IL-1α, IL-1β, and TNFα, were increased in the colon of both WT and IL-17KO mice after DSS treatment. However, somewhat unexpectedly, expression levels of IL-1α and TNFα were lower in the colons of IL-17KO mice. Moreover, IL-6 mRNA was upregulated only in inflamed colons of WT mice. The expression level of mRNA for IL-17, but not its related cytokine IL-17F, was increased in colon of WT mice after DSS treatment.

### Aggravated colitis seen in IL-17KO mice correlates with the lack of CD11b^+^Ly6c^+^MHC Class II^+^ macrophages

**Figure 3:**
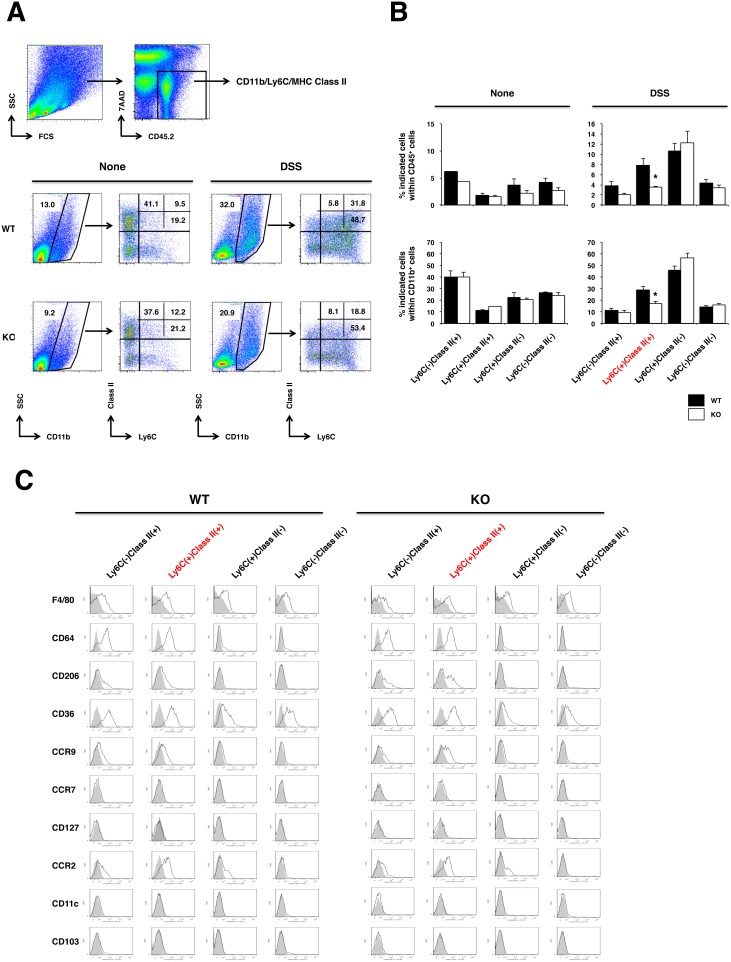
Aggravated DSS-induced colitis in IL-17KO mice is associated with reduced number of CD11b^+^Ly6C^+^MHC Class II^+^ macrophages in the inflamed colon. IL-17KO mice and WT controls were treated with DSS as described in the legend for Figure 1. (A) LPLs were purified from pooled (n = 2–3) distal colon sections of untreated and DSS-treated mice and subjected to flow cytometry analysis. Gating strategy of live and CD45^+^ for CD11b^+^ cells was shown in upper panels. Representative data from seven independent experiments are shown. Number denotes frequency of gated cells. (B) The frequency of cells for each subset in A is shown. Graphs represent mean ± SEM of seven independent experiments. *p<0.05. (C) Representative flow cytometry profiles of cell surface molecules implicated in M2/anti-inflammatory macrophages on each subset depicted as in A. Gray histograms represent cells stained with isotype matched control mAbs.

Intestinal macrophages are highly versatile in function and can suppress inflammation and/or promote repair of damaged mucosal tissues [23]. Together with our results that genes involved in anti-inflammatory/tissue repair, which expressed in M2/wound healing macrophages were reduced in the inflamed colon of IL-17KO mice mentioned above, led us to examine for differences between macrophage subpopulations in the inflamed colon of WT and IL-17KO mice. As an initial step, we performed multi-color flow cytometric analysis for mononuclear cells of colonic lamina propria taken from IL-17KO and WT mice before and after induction of colitis. At a steady state, there is only a small difference in the proportion of CD11b^+^ cells, and subsets within CD11b^+^ cell population of colonic lamina propria leukocytes (LPLs) from IL-17KO and WT mice (Figure 3A and B). Inflamed colonic LPLs contained an increasing trend in the proportion of CD11b^+^ cell infiltrates but this increase was less prominent in IL-17KO mice (Figure 3A). On the other hand, there was a significant difference in the proportion of Ly6C^+^MHC Class II^+^ cells between WT and IL-17KO mice within CD11b^+^ cell population (Figure 3B). Inflamed colonic LPLs of IL-17KO mice contained a significantly lower proportion of Ly6C^+^MHC Class II^+^ cells. As for other cell types involved in the regulation of mucosal immune responses, there was a notable decrease in frequency of CD4^+^Foxp3^+^ regulatory T cells in the inflamed colon LPLs of IL-17KO mice (Figure S1). We then looked at expression levels of CD206, CD36 and CCR9 along with F4/80 and CD64, all of which are associated with macrophages bearing anti-inflammatory and/or tissue repair function [20], [23], [24], on individual subpopulations as defined by Ly6C and MHC Class II within CD11b^+^ cells (Figure 3C). Ly6C^+^MHC Class II^+^ and Ly6C^−^MHC Class II^+^ subpopulations expressed higher levels of those molecules as compared to Ly6C^+^MHC Class II^−^ and Ly6C^−^MHC Class II^−^ subpopulations in inflamed colonic LPLs of WT and IL-17KO mice. Consistent with these findings, immunohistochemical analysis of colonic tissue sections showed that the inflamed colons of WT mice enriched Ly6C^+^MHC Class II^+^ and Ly6C^+^CD206^+^ cells, but these features were less prominent in IL-17KO mice (data not shown). As for cell surface markers, such as CCR7, CD127, CD11c, and CD103 implicated in M1 macrophages and dendritic cells, both of which have been shown to play proinflammatory role in colitis [20], [23], [25] were not expressed on either subpopulations within CD11b^+^ cells (Figure 3C).

### CD11b^+^ cells in the inflamed colonic LPLs of WT mice express signature transcription factors and their target genes implicated in anti-inflammatory M2/wound healing macrophages

**Figure 4:**
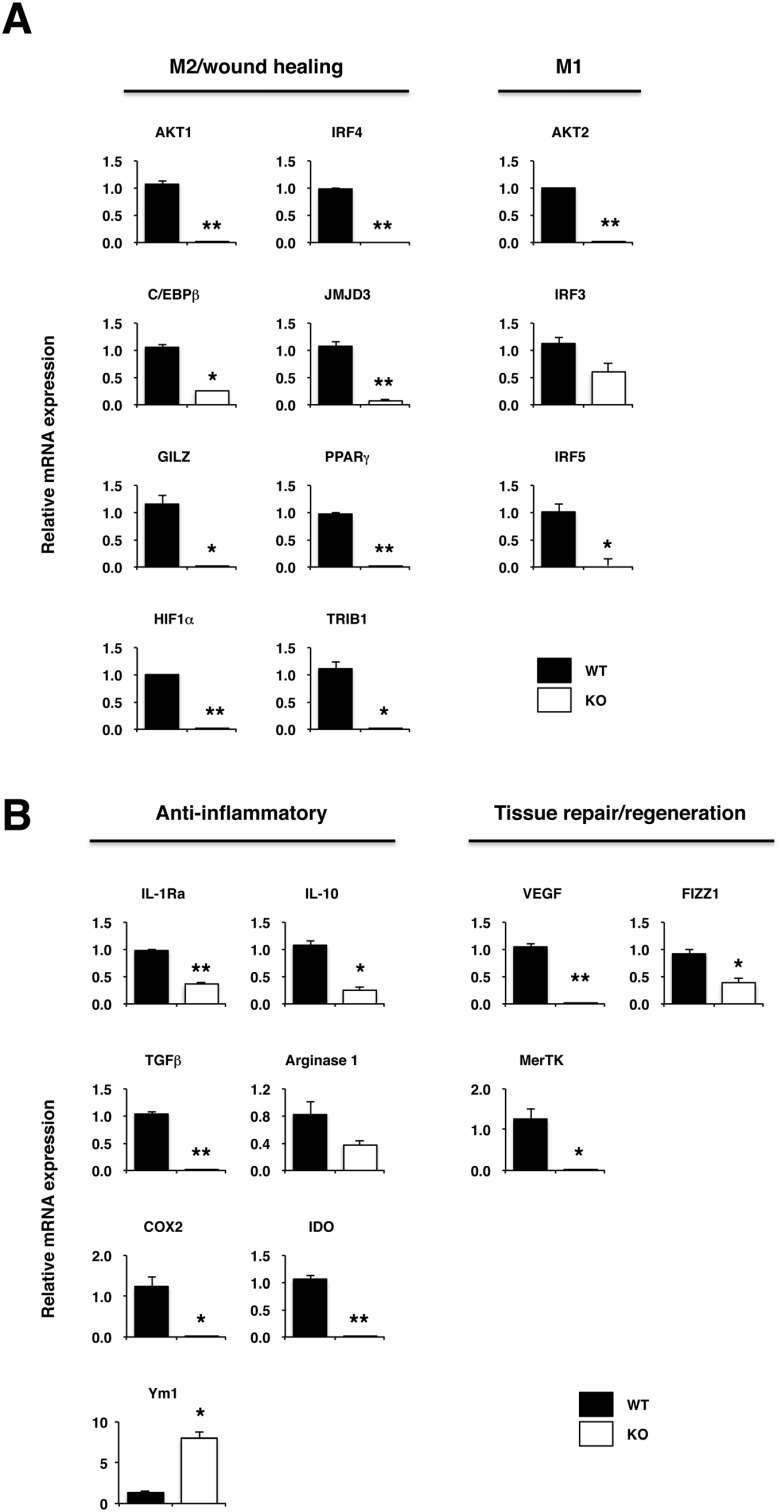
CD11b^+^ cells from inflamed colonic LPLs of KO mice express significantly lower levels of genes implicated in M2/wound healing macrophages. IL-17KO mice and WT controls (n = 3) were treated with DSS as described in the legend for Figure 1. Then, LPLs isolated from these mice were further purified into CD11b^+^ cell on FACS Aria, from which cDNA were prepared and subjected to real-time PCR analysis. Expression of target mRNA were normalized to the expression of β-actin mRNA for generation of ΔCt values, and relative mRNA expression was quantified with the ΔΔCt method. Data are expressed as mean ± SEM. *p<0.05; **p<0.01.

To gain insight into the unique features of macrophages that emerged in the inflamed colons of WT that seem to have regulatory function for colitis, we looked into genes expressed in CD11b^+^ cells, more abundant in Ly6C^+^MHC Class II^+^ cell in LP of colitic WT mice as compared to those from IL-17KO mice. To this end, CD11b^+^ cells from LP of colitic WT and IL-17KO mice were purified by FACS (Figure S2), and their mRNAs were subjected to real-time PCR analysis. As shown in Figure 4A, transcription factors implicated in polarization of M2 and/or M2-like wound healing macrophages [26] were higher in CD11b^+^ cells from LPLs of WT mice as compared to KO mice. Accordingly, increased expression of genes coding for anti-inflammatory functions, such as IL-1Ra, IL-10, TGFβ, arginase 1, COX2, and IDO, and those for wound healing functions, such as VEGF, FIZZ1, and MerTK, were upregulated in CD11b^+^ of WT mice (Figure 4B) [20]. Somewhat surprisingly, expression levels of mRNA for transcription factors linked to proinflammatory M1 macrophages, such as AKT2, IRF3, and IRF5 were also higher in CD11b^+^ macrophages from WT mice (Figure 4A). Inversely, the expression level of Ym-1, one of a maker for M2 macrophages with anti-inflammatory function, was lower in CD11b^+^ macrophages from WT mice (Figure 4B).

### Extrinsic IL-17 induces differentiation of blood monocytes into CD11b^+^Ly6C^+^MHC Class II^+^ macrophages in the inflamed colon

**Figure 5:**
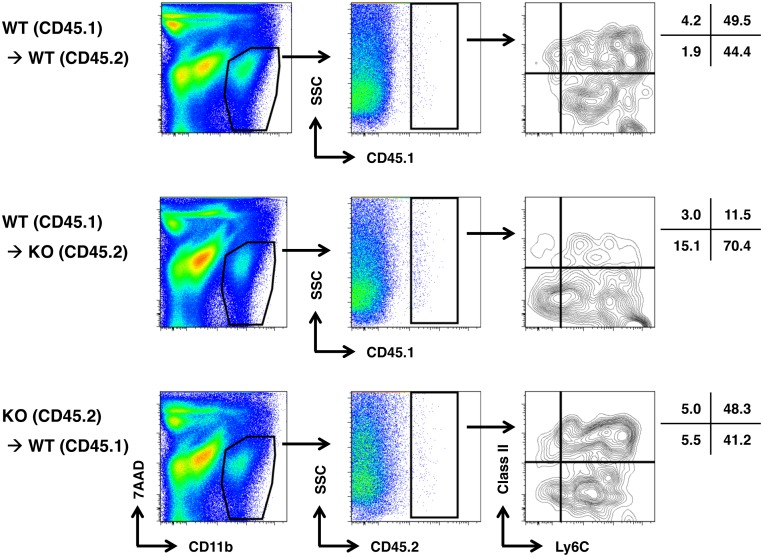
Blood monocytes are recruited into inflamed colons and differentiate into CD11b^+^Ly6C^+^MHC Class II^+^ macrophages in the presence of IL-17. Monocytes were purified from bone marrow of WT (CD45.1), WT (CD45.2) or IL-17KO (CD45.2), and adaptively transferred into WT (45.2), IL-17KO (CD45.2) or WT (CD45.1) mice (n = 3), respectively, followed by the treatment with DSS. On day 10, colonic LPLs were isolated from pooled colon, stained with anti-CD11b, anti-Ly6C, and anti-MHC Class II together with corresponding anti-CD45 congenic antibody, and subjected to flow cytometry analysis. A representative result from three independent experiments is shown.

Based on the finding that the expression level of CCL2 mRNA was increased in the inflamed colons and CCR2 was expressed on Ly6C^+^MHC Class II^+^ cells within CD11b^+^ cells (Figure 2 and 3C), we next sought to determine whether CCR2^+^Ly6C^+^ blood monocytes are recruited predominantly to the inflamed colon and differentiate in situ under the influence of IL-17 into CD11b^+^Ly6C^+^MHC Class II^+^ macrophages. To this end, monocytes were adoptively transferred into IL-17 sufficient or deficient mice, which received DSS treatment thereafter. On day 10, LPLs were harvested from these mice, among which donor cells were identified based on congenic markers and evaluated for their cell surface phenotype. As a representative result shown in Figure 5, most of donor monocytes, regardless of whether they were from WT or IL-17KO mice, gained MHC Class II expression and maintained Ly6C expression in WT host, whereas only a fraction of them gained MHC Class II in IL-17KO host. These results suggest that IL-17 is involved in the phenotypic and possibly functional maturation of monocytes by extrinsic mechanism.

### Depletion of CD11b^+^Ly6C^+^MHC Class II^+^ macrophages exacerbates colon inflammation induced by DSS

**Figure 6:**
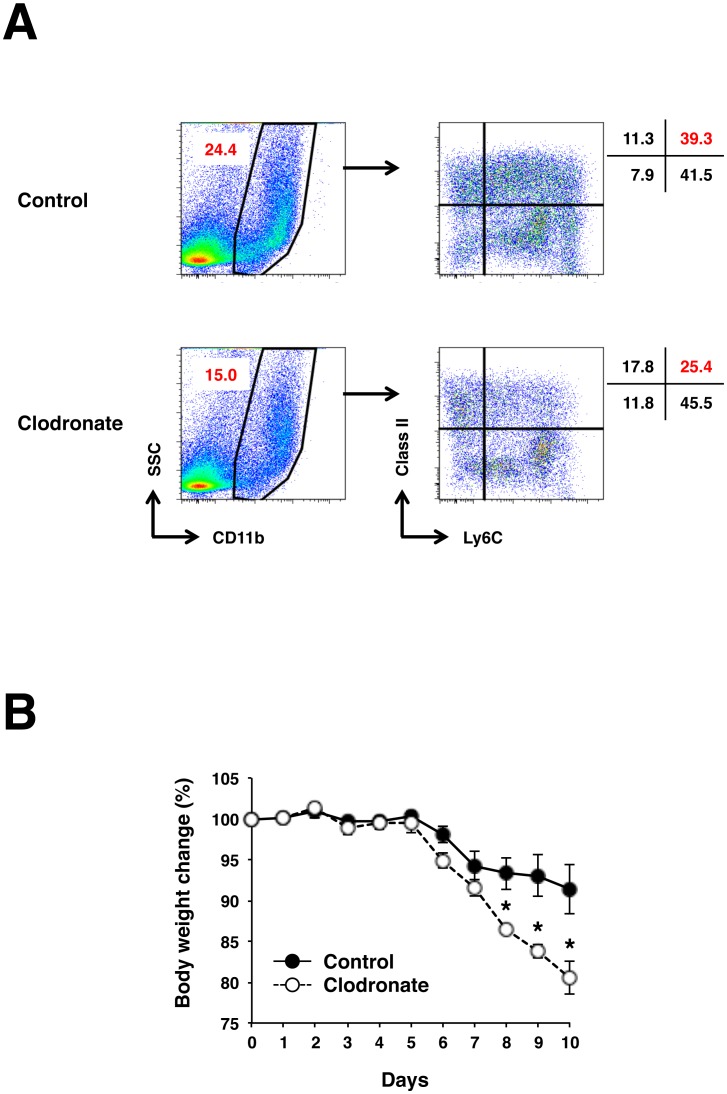
Depletion of CD11b^+^Ly6C^+^MHC Class II^+^ macrophages accelerates colon inflammation in WT mice induced by DSS treatment. WT mice were given 1.5% DSS in drinking water for 7 days followed by consumption of water alone for another 3 days, during which the mice were treated with clodronate-liposome or control liposome intrarectally on days −1, 1, 3, and 5 as described in Materials and Methods. (A) Representative FACS plots showing reduced CD11b^+^Ly6C^+^MHC Class II^+^ macrophages in colon of clodronate-liposome treated mice. (B) Changes in body weight over time were expressed as a percentage of the original weight. Data represent as mean ± SEM. The experiments were repeated two times with at least three mice per group per experiment.

Having confirmed that IL-17KO mice suffering more severe colitis had impaired ability to generate Ly6C^+^MHC Class II^+^ cells expressing the highest M2 marker within CD11b^+^ cell population and CD11b^+^ cells of the inflamed colonic LPLs from WT mice abundant in Ly6C^+^MHC Class II^+^ cells expressed increased level of most genes implicated in M2/wound healing macrophages (Figure 3 and 4), we next sought to determine whether CD11b^+^Ly6C^+^MHC Class II^+^ cells are responsible for reduced colonic inflammation seen in WT mice. Liposome uptake by macrophages represents a genuine phagocytosis event [27], which has been widely used to target macrophage in vivo. Taking advantage of the fact that M2 macrophages have a high phagocytic activity [28] and intrarectal administration of clodronate-liposome, we could preferentially decrease Ly6C^+^MHC Class II^+^ over other subpopulations in LPLs, but not systemically, of DSS treated WT mice (Figure 6A and Figure S3). This treatment reduced colonic CD11b^+^ cells by 35.6% (27.5±0.9% → 17.7±0.8%, p<0.01) and Ly6C^+^MHC Class II^+^ cells within CD11b^+^ population by 35.4% (33.4±1.5% → 24.0±0.5%, p<0.01), resulting in an overall decrease in CD11b^+^ Ly6C^+^MHC Class II^+^ macrophages by 53.9% (9.1±0.2% → 4.2±0.1%, p<0.01) in five independent experiments. On the other hand, mononuclear phagocyte populations as defined by CD11b, Ly6C and MHC Class II were marginally, if at all, affected in PBMC, spleen and bone marrow by this treatment (Figure S3). As expected, WT mice treated with clodronate-liposome exhibited a greater degree of body weight loss as compared to the mice treated with control-liposome (Figure 6B). Taken as a whole, these results indicate that CD11b^+^Ly6C^+^MHC Class II^+^ macrophages differentiated from blood monocytes in the presence of IL-17 play a regulatory role in colonic inflammation.

## Discussion

IL-17 has a critical function in the host defense response against various pathogens, but also has become notorious for its role in the pathogenesis of many inflammatory and autoimmune disorders, which makes this cytokine categorized as a proinflammatory cytokine [3]. This prevailing view was also adopted in IBD where IL-17 and IL-17 producing cells were found abundantly in the affected tissue [4]. Indeed, numerous studies have demonstrated pro-colitogenic role of IL-17 in animal models of IBD [8]–[10], [12]. However, challenging this view, other studies in animal models [11], [13], [14] and recent human clinical trials [29], [30] have emerged to suggest that IL-17 plays a protective role. To reassess the role of IL-17 in IBD pathogenesis and underlying mechanisms involved, we adopted a well-know DSS-induced colitis model, namely WT or IL-17KO mice were given DSS in drinking water. Upon evaluation of ensuing colitis, we found that IL-17KO mice were much more susceptible than WT mice. In addition, expression levels of mRNA coding for most, but not all, of the molecules contributing to suppression/resolution of inflammation and tissue repair [20], [31] were significantly reduced in the inflamed colons of IL-17KO mice. Among those, genes expressed by M2/wound healing macrophages were downregulated in IL-17KO mice with notable consistency.

A characteristic of an inflammatory landscape in the colon of patients with IBD is an increased number of macrophages derived from blood monocytes [1], [2]. As compared to a healthy colon, these macrophages produce an increased amount of inflammatory cytokines and express cell surface molecules involved in the activation of their own and T cells [1], [2]. In a mouse model of colitis, these macrophages are CD11b^+^F4/80^+^MHC Class II^+^CX3CR1^int^ driving inflammation through various effector mechanisms [25], [32]. Hence, aberrant activation and functions of intestinal macrophages have been proposed to contribute to the IBD pathogenesis. However, recent studies also indicate that macrophages are functionally highly promiscuous, some of which also produce factors that dampen inflammatory responses while facilitating tissue repair [20], [23], [33]. Indeed, anti-TNF therapy for patients with IBD induces macrophages with regulatory functions, which promote wound healing [34]. In an animal model of colitis, macrophages have also been shown to exert disease ameliorating effects. These macrophages include CD11b^+^F4/80^+^MHC Class II^+^ cells coexpressing CD11c and/or also CX3CR1 [35], [36]. It has also been shown that they are recent emigrant from blood monocytes [37]. Recent study also points that CD64 as a specific macrophage marker that can discriminate dendritic cells from macrophages in the murine intestine under both steady-state and inflammatory conditions [24]. In our present study, we found a significant decrease in the frequency of CD11b^+^Ly6C^+^MHC Class II^+^CD64^+^ macrophages, which were derived, at least in part, from blood monocytes, in the inflamed colon of IL-17KO mice as compared to WT mice. Although statistically non-significant due to the variation in number of cells recovered from LPLs in each experiment, the absolute numbers of CD11b^+^Ly6C^+^MHC Class II^+^ macrophages were almost consistently reduced in IL-17KO mice. Furthermore, depletion of this population by topical administration of clodronate-liposome resulted in the exacerbation of DSS-induced colitis in WT mice, clearly indicating that these macrophages ameliorate, rather than exacerbate, colitis. In support of this notion, recent studies have shown that CD11b^+^Ly6C^+^MHC Class II^+^ macrophages in inflamed intestine produce both inflammatory and anti-inflammatory molecules to control invading pathogen while limiting collateral tissue damage [38], [39]. Commercially available Abs do not allow to detect CX3CR1 by flow cytometry or immunohistochemistry, we were unable to determine whether CD11b^+^Ly6C^+^MHC Class II^+^ macrophages express CX3CR1 and belong to the same or overlapping population mentioned above, an important issue which needs to be further investigated.

CD11b^+^ cells isolated from the inflamed colons of WT mice enriched in CD11b^+^Ly6C^+^MHC Class II^+^ macrophages expressed higher levels of mRNA encoding anti-inflammatory and tissue repair functions as compared to CD11b^+^ cells from IL-17KO mice. A discrepancy remains, however, in that CD11b^+^ cells from the inflamed colons of IL-17KO mice expressed higher levels of Ym1 mRNA, which is a marker for M2 macrophages [40]. Resent study indicates that GM-CSF is critical in the expression of Ym1 [41]. Therefore, the higher levels of GM-CSF in the inflamed colons of IL-17KO mice as compared to WT mice (data not shown) may explain this seemingly paradoxical observation. Taken as a whole, it is possible that through the production of factors with anti-inflammatory/tissue repair functions, CD11b^+^Ly6C^+^MHC Class II^+^ macrophages suppress inflammation while quickly repairing colon tissue before serious damage to the tissue occurs, resulting in less severe colitis seen in WT mice. Recent studies also suggest that a subset of macrophages in the colon play a crucial role in the maintenance and/or promotion of differentiation of functional Foxp3^+^ regulatory T cells. Together with our present results that inflamed colon of IL-17KO mice contained reduced levels of Foxp3^+^ T cells, it is also possible that CD11b^+^Ly6C^+^MHC Class II^+^ macrophages ameliorate colitis through enhancement of Foxp3^+^ regulatory T cell function [23]. In support of this notion, we observed that the depletion of CD11b^+^Ly6C^+^MHC Class II^+^ macrophages in colon of WT mice by clodronate-liposome was associated with the reduction in Foxp3^+^CD4^+^T cells by 36% (30.3±0.63% → 19.2±0.62%, p<0.01, n = 4).

Perhaps more importantly, our study revealed that monocytes differentiate to express molecules and genes implicated in M2/wound healing macrophages under the influence of IL-17 in the inflamed colon. In support of this notion, recent studies show that IL-17 stimulates differentiation of M2/anti-inflammatory macrophages [42], [43]. However, in the present study we showed that along with elevated expression of mRNA for M2 associated transcription factors and anti-inflammatory/tissue regenerative factors, CD11b^+^ myeloid cells in the inflamed colons of WT mice also expressed higher levels of mRNA for M1 associated transcription factors. Thus, macrophages differentiated under the influence of IL-17 did not fit comfortably with the M1/M2-paradigm of differentiated macrophages. We speculate that macrophages may acquire the unique M2 dominant properties adapted to the inflamed colon microenvironment under the influence of a unique cytokine milieu involving IL-17.

In summary, our study shows, for the first time, that CD11b^+^Ly6C^+^MHC Class II^+^ macrophages differentiated in the inflamed colon under the influence of IL-17 represent M2-like/wound healing macrophages which may have regulatory functions. Whether the induction of this population by IL-17 solely explains all of the protective function of IL-17 in colitis remains arguable, since we also observed that inflamed colonic tissue of IL-17KO mice expressed reduced levels of claudin-1/2, β-difensin-1/2, and mucin-2, all of which have been shown to be regulated by IL-17 signaling. However, our data demonstrate a previously unappreciated mechanism by which IL-17 exerts protective functions in colitis and targeting IL-17/M2-like macrophage axis may represent an important future therapeutic approach in the treatment of mucosal inflammatory diseases such as IBD.

## Supporting Information

Figure S1 **Flow cytometric analysis of LPL from WT and IL-17KO mice.** IL-17KO mice and WT controls were given 1.5% DSS in drinking water for 7 days followed by consumption of water alone for another 3 days. LPLs were purified from pooled (n = 2–3) distal colon sections of untreated and DSS-treated mice and subjected to flow cytometry analysis after staining with mAbs specific for indicated cell surface and intracellular molecules. Plots were shown after electric gating for 7AAD^−^ and CD45^+^ cells. Number denotes frequency of gated cells. Representative results of at least two independent experiments are shown.
(TIF)

Figure S2 **Flow cytometry analysis of CD11b^+^ cells before and after cell sorting.** IL-17KO mice and WT controls were given 1.5% DSS in drinking water for 7 days followed by consumption of water alone for another 3 days. LPLs were purified from pooled (n = 10) distal colon sections of untreated and DSS-treated mice followed by cell sorting by FACS. Representative results out of two independent experiments are shown.
(TIF)

Figure S3 **Representative flow cytometry plots on CD11b^+^Ly6C^+^MHC Class II^+^ macrophage population in organs other than colon of WT mice treated with clodronate-liposome.** WT mice were given 1.5% DSS in drinking water for 7 days followed by consumption of water alone for another 3 days, during which the mice were treated with clodronate-liposome or control liposome intrarectally on days −1, 1, 3, and 5 and their body weight changes were monitored. The experiments were repeated two times with at least tree mice per group per experiment.
(TIF)

Table S1 **Primer sequences used for realtime RT-PCR.**
(DOCX)
