# Supplementary figures and images for: Interleukin-17 Induces an Atypical M2-Like Macrophage Subpopulation That Regulates Intestinal Inflammation
Source: PLoS One. 2014 Sep 25;9(9):e108494. doi: 10.1371/journal.pone.0108494 (PMC4177893; doi:10.1371/journal.pone.0108494)

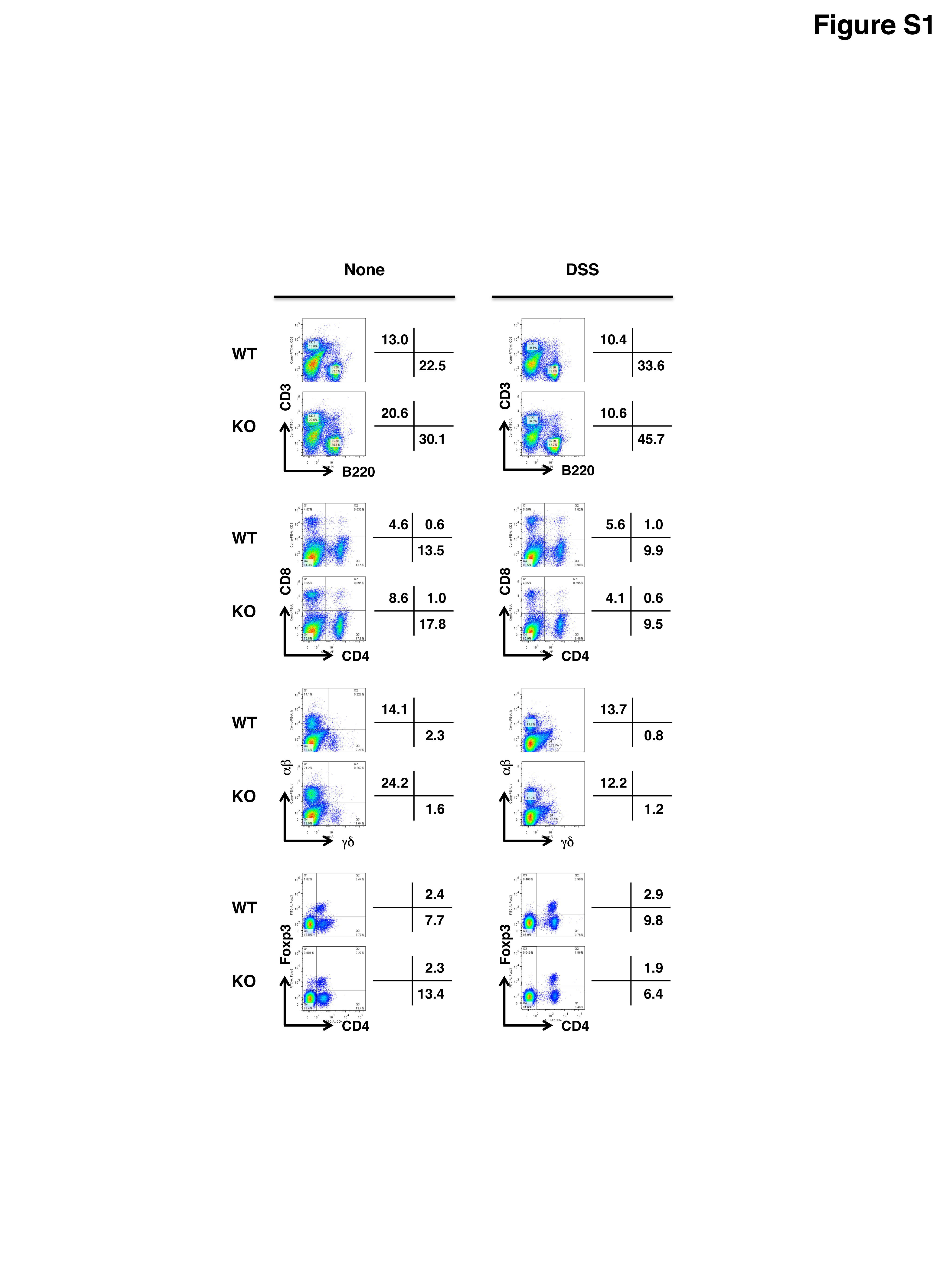

Supplement: Figure S1 — Flow cytometric analysis of LPL from WT and IL-17KO mice. IL-17KO mice and WT controls were given 1.5% DSS in drinking water for 7 days followed by consumption of water alone for another 3 days. LPLs were purified from pooled (n = 2–3) distal colon sections of untreated and DSS-treated mice and subjected to flow cytometry analysis after staining with mAbs specific for indicated cell surface and intracellular molecules. Plots were shown after electric gating for 7AAD− and CD45+ cells. Number denotes frequency of gated cells. Representative results of at least two independent experiments are shown. (TIF) [file pone.0108494.s001.tif]

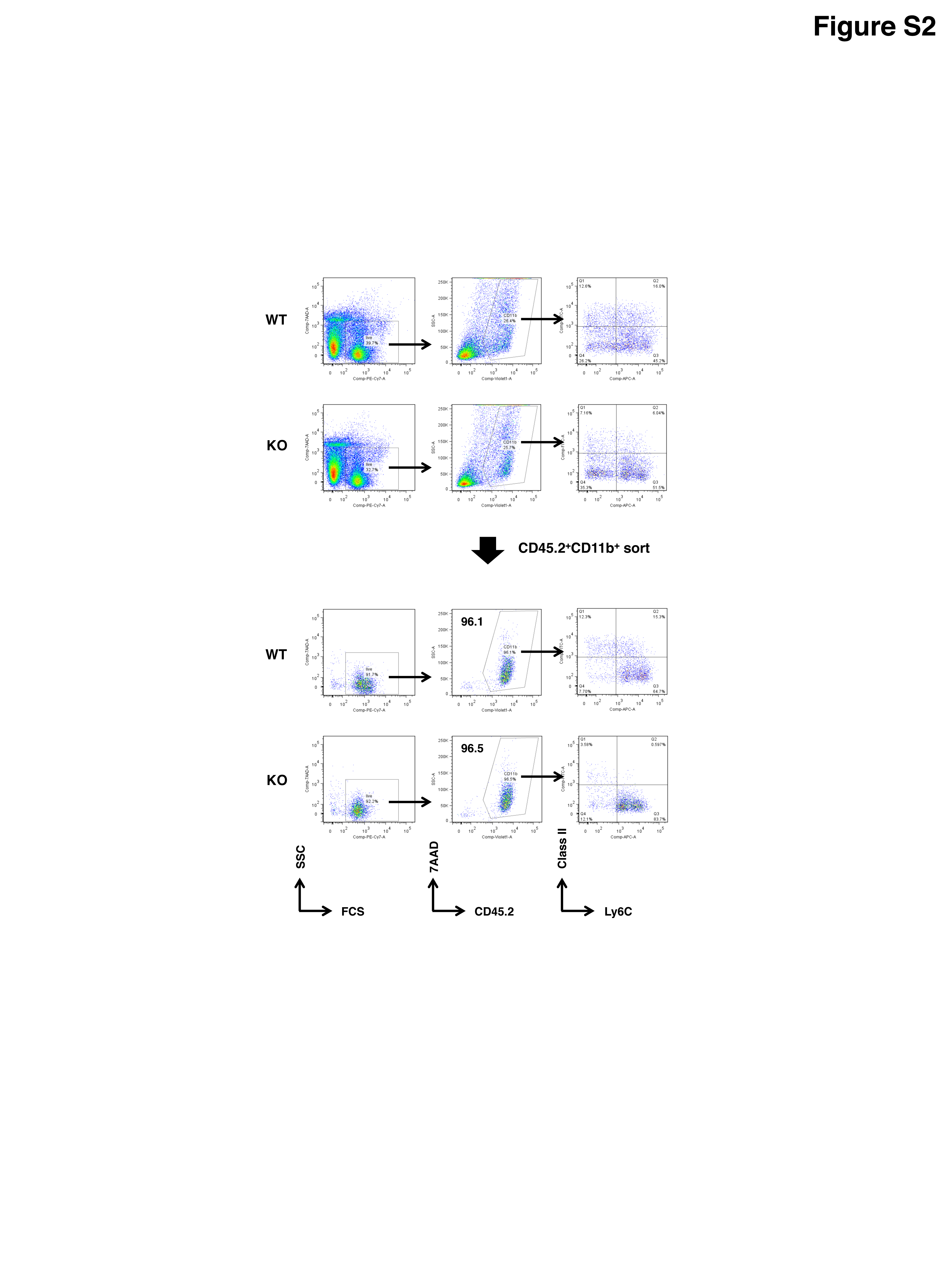

Supplement: Figure S2 — Flow cytometry analysis of CD11b+ cells before and after cell sorting. IL-17KO mice and WT controls were given 1.5% DSS in drinking water for 7 days followed by consumption of water alone for another 3 days. LPLs were purified from pooled (n = 10) distal colon sections of untreated and DSS-treated mice followed by cell sorting by FACS. Representative results out of two independent experiments are shown. (TIF) [file pone.0108494.s002.tif]

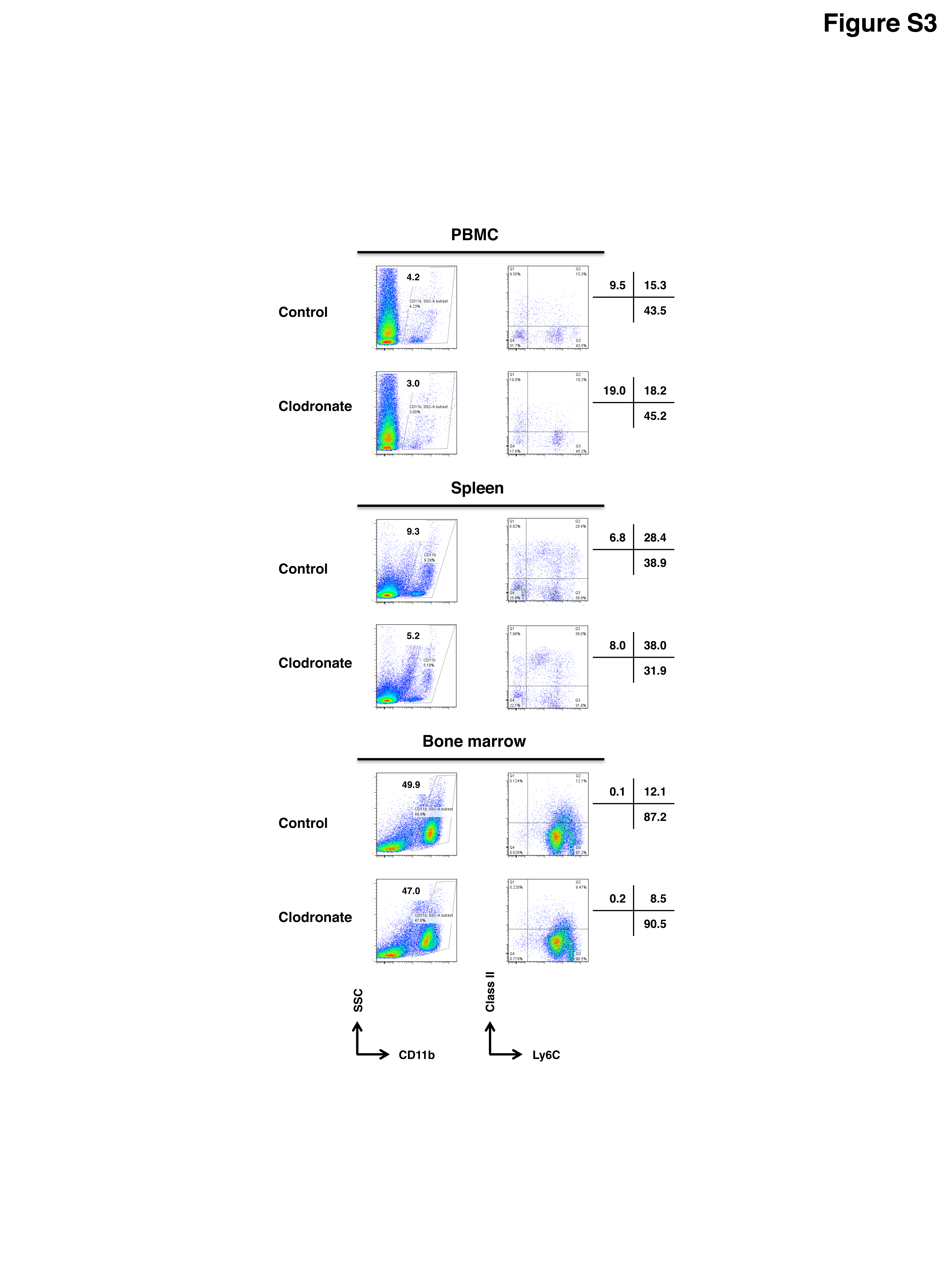

Supplement: Figure S3 — Representative flow cytometry plots on CD11b+Ly6C+MHC Class II+ macrophage population in organs other than colon of WT mice treated with clodronate-liposome. WT mice were given 1.5% DSS in drinking water for 7 days followed by consumption of water alone for another 3 days, during which the mice were treated with clodronate-liposome or control liposome intrarectally on days −1, 1, 3, and 5 and their body weight changes were monitored. The experiments were repeated two times with at least tree mice per group per experiment. (TIF) [file pone.0108494.s003.tif]
