# Supplementary material for: Interleukin-17 Induces an Atypical M2-Like Macrophage Subpopulation That Regulates Intestinal Inflammation
Source: PLoS One. 2014 Sep 25;9(9):e108494. doi: 10.1371/journal.pone.0108494 (PMC4177893; doi:10.1371/journal.pone.0108494)
Supplement: Table S1 — Primer sequences used for realtime RT-PCR. (DOCX) [file pone.0108494.s004.docx]

| **Table S1. Primer sequences for RT-PCR** | | |
| --- | --- | --- |
| **GENES** | **FORWARD PRIMER** | **REVERSE PRIMER** |
| **Akt1** | TGAGGAGCGGGAAGAATGG | GGTGAGCCTGATCGGAAGTC |
| **Akt2** | GCCGGTGACAGACGATACTGT | TTCACACGCTGTCACCTAGCTT |
| **Arg1** | CTGCATGGGCAACCTGTGT | CCTGGTACATCTGGGAACTTTCC |
| **Actb** | GCTCTGGCTCCTAGCACCAT | GCCACCGATCCACACAGAGT |
| **Ccbp2** | AACCAGCAATCCGCAGCTA | CTCGAGCTCTGATGCTTGCA |
| **Cebpb** | CGCAACACACGTGTAACTGTCA | AACAACCCCGCAGGAACAT |
| **csf1** | GCAGCAGTTGATCGACAGTCA | CGCATGGTCTCATCTATTATGTCTTG |
| **csf2** | GCCATCAAAGAAGCCCTGAAC | CGACTTCTACCTCTTCATTCAACGT |
| **Retnla** | CCTGCTGGGATGACTGCTACT | TCCACTCTGGATCTCCCAAGA |
| **Tsc22d3** | GCCATGGACCTCGTGAAGA | TTTAGGACCTCCACCTCCTCTCT |
| **Hif1a** | GCGGGCACCGATTCG | TTCAGAACTCATCTTTTTCTTCTCGTT |
| **Ido1** | GAGAAAGCCAAGGAAATTTTTAAGAG | CGGAGAACGTGGAAAAACGT |
| **Ifng** | GCAACAGCAAGGCGAAAAAG | CTGGACCTGTGGGTTGTTGAC |
| **Il10** | CATTTGAATTCCCTGGGTGAGA | GTAGACACCTTGGTCTTGGAGCTTA |
| **Il13** | GCCGGTGCCAAGATCTGT | GGGAGTCTGGTCTTGTGTGATG |
| **Il17a** | TGAGGACTCGCAAACATG | GCAGCAACAGCATCAGAGACA |
| **Il17f** | CCCCATGGGATTACAACATCA | CACTGGGCCTCAGCGATCT |
| **Il1a** | GAGTCGGCAAAGAAATCAAGATG | AACTGTAGTCTTCGTTTTCACTGTAACAG |
| **Il1b** | TGACAGTGATGAGAATGACCTGTTC | TTGGAAGCAGCCCTTCATCT |
| **Il1rn** | CCAGCTGGAGGAAGTTAACATCA | TCAGAGCGGATGAAGGTAAAGC |
| **Il22** | CAGCAGCCGTACATCGTCAA | TCTGCAAGGCTGACCTCCTT |
| **Il4** | CGATGCCTGGATTCATCGATA | TTTCCAGGAAGTCTTTCAGTGATGT |
| **Il6** | CGTGGAAATGAGAAAAGAGTTGTG | TCCTGATTATATCCAGTTTGGTAGCAT |
| **Irf3** | TTCGTGGCAGATCTGATTGC | CCATGCAGAACCACAGAGTGTAG |
| **Irf4** | CGCATCCCGTGGAAACAC | ACAATGCCCAAGCCTTGAAG |
| **Irf5** | TTTGAGATCTTCTTTTGCTTTGGA | GTACCACCTGTACAGTAATGAGCTTCTT |
| **Kdm6b** | CGAGTGGTTCGCGGTACAT | GAATATTGGACGCATAGAGGTCATC |
| **Mertk** | CAGCATTCAGGTCAAGGAAGCT | TGTGGCGAAGCAGAGGTATTAA |
| **Pparg** | CACAAGAGCTGACCCAATGGT | GATCGCACTTTGGTATTCTTGGA |
| **Ptgs2** | CAGCCAGGCAGCAAATCC | TTATACTGGTCAAATCCTGTGCTCAT |
| **Reg3b** | GGCTCCTACTGCTATGCCTTGT | CAGGCCAGTTCTGCATCAAA |
| **Reg3g** | TTTCTCAGGTGCAAGGTGAAGTT | GGGCAGCTGCTACGTGAAG |
| **Tgfb1** | GCCCTTCCTGCTCCTCATG | GCAGTTCTTCTCTGTGGAGCTGA |
| **Tnfa** | GACCCTCACACTCAGATCATCTTCT | TCCACTTGGTGGTTTGATACG |
| **Trib1** | GCTGCAAGGAATTCCCCATT | GACGGCAGCTGGATGTAAGG |
| **Table S1. Primer sequences for RT-PCR (continued)** | | |
| **GENES** | **FORWARD PRIMER** | **REVERSE PRIMER** |
| **Vegf** | AAGCCAGCACATAGGAGAGATGA | CTGTCTTTCTTTGGTCTGCATTCA |
| **Chi3l3** | TCTGGTGAAGGAAATGCGTAAA | GCAGCCTTGGAATGTCTTTCTC |
| **Tslp** | GGCTACCCTGAAACTGAGAGAAAT | TGATTCAGGCAGATGTTTTGGA |
